# Crosstalk between the Ketogenic Diet and Epilepsy: From the Perspective of Gut Microbiota

**DOI:** 10.1155/2019/8373060

**Published:** 2019-06-03

**Authors:** Yuying Fan, Hua Wang, Xueyan Liu, Junmei Zhang, Gang Liu

**Affiliations:** ^1^Department of Pediatrics, Shengjing Hospital of China Medical University, Shenyang, Liaoning 110004, China; ^2^Hunan Provincial Key Laboratory of Animal Nutritional Physiology and Metabolic Process, CAS Key Laboratory of Agro-ecological Processes in Subtropical Region, Institute of Subtropical Agriculture, Chinese Academy of Sciences, Changsha, Hunan 410125, China

## Abstract

Given the association between a range of neurological disorders and changes in the gut microbiota, interest in the gut microbiota has recently increased. In particular, the significant involvement of the autoimmune processes in the development of epilepsy, one of the most serious and widespread neurological diseases, has led to a suggested link with the gut microbiome. Because the constitution of the gut microbiome can be influenced by diet, dietary therapy has been shown to have a positive impact on a wide range of conditions via alteration of the gut microbiota. An example of one such diet is the ketogenic diet (KD), which promotes a diet that contains high levels of fat, adequate levels of protein, and low levels of carbohydrate. Due to the near-total elimination of carbohydrates from the individual's food in this ultra-high-fat diet, ketone bodies become an important source of energy. Although the ketogenic diet has proven successful in the treatment of refractory epilepsy and other illnesses, the underlying mechanisms of its neuroprotective effects have yet to be fully elucidated. Nevertheless, recent studies strongly indicate a role for the gut microbiota in the effective treatment of epilepsy with the ketogenic diet. The latest advances regarding the links between the ketogenic diet, gut microbiota, and epilepsy are reviewed in this article, with a particular focus on the role of the gut microbiota in the treatment outcome.

## 1. Introduction

The diverse population of microbes in the gastrointestinal tract, including archaea, bacteria, fungi, protozoa, and viruses, is generally referred to as the gut microbiota [1]. Investigation of the role of the gut microbiota in disease and health has recently attracted increasing interest, and a growing body of evidence suggests a role for the gut microbiota in a wide range of neurological disorders via gut-brain interactions [2–5].

Epilepsy is among the most serious and widespread neurological disorders and represents a major liability for the healthcare system [6]. Moreover, approximately one-third of epilepsy patients eventually develop drug resistance, defined as the failure to achieve lasting freedom from seizures after sufficient trials with two tolerated, correctly selected, and administered antiepileptic drugs, either as a single therapy or as part of a combined treatment regimen [7–9].

Significant involvement of autoimmune processes in the development of epilepsy has been noted in a growing body of research [10–12]. Because microbiota are also strongly correlated with autoimmunity [13, 14], it is plausible that the specific composition of the gut microbiota population could influence both the individual's susceptibility to epilepsy and the subsequent progression of the illness [15, 16].

A number of studies on epilepsy and microbiota have recently been published, and many have demonstrated the significant impact of diet on the composition of the gut microbiota and on the subsequent health of the individual [17, 18]. The high-fat, sufficient-protein, and very low-carbohydrate ketogenic diet (KD) was established early in the 1920s to reproduce the central metabolic impacts and associated antiseizure effects of fasting [19]. The KD has since found continued world-wide use in the treatment of drug-resistant epilepsy [20, 21]. The function of the gut microbiota in the treatment of epilepsy with the KD has recently been examined, and some recent studies have indicated that the KD alters the gut microbiota of individuals in the diseased state [22]. In this review, the latest advances regarding the crosstalk among the ketogenic diet, the gut microbiota, and epilepsy are reviewed with a focus on the role of the gut microbiota.

## 2. Microbiota and Epilepsy

### 2.1. Microbiota-Brain Axis

The gut microbiota is a multifaceted ecological population that contains trillions of microorganisms that inhabit the animal digestive tract and exhibits a range of dynamic interactions with the host's immune system and assists in maintaining the metabolic dynamic equilibrium [23–25]. Rather than being a merely commensal relationship, the association between the host organism and the gut microbiota is a mutualistic symbiosis [26]. Interest in the function of the gut microbiota in brain disorders has recently increased, with studies indicating that alterations in the gut microbiota could influence the brain's behavioural, cognitive, and physiological functions [27–30]. Although the precise mechanism of crosstalk between the brain and the gut microbiota has yet to be fully elucidated, the effect of the microbiota on the brain has become a hot topic within the field of neuroscience [27–29]. Broadly speaking, the gut microbiota influences the brain via a number of pathways, including the endocrine, immune, and metabolic systems, in addition to the neuroanatomical route between the gut and the brain provided by the nervous system [31–34]. The two-way communication link between the gut and brain is termed the gut-brain axis, whilst the interaction between the gut microbiota and the gut-brain axis is termed the gut microbiota-gut-brain axis or the gut microbiota-brain axis [35]. There has been significant focus on the function of microbes in the gut microbiota-brain axis because the gut microbiota can be deliberately altered, thereby providing an independent variable that can be therapeutically manipulated [36].

### 2.2. Gut Microbiota and Epilepsy

#### 2.2.1. Epilepsy Correlates with Gut Microbiota through Autoimmunity

Epilepsy is a widespread serious and chronic neurological disorder that can severely delay development, reduce brain function, and lead to a significant mortality rate [37, 38]. Approximately one-third of epileptic individuals are resistant to drugs and thus extremely difficult to treat [39]. Although the aetiology of epilepsy has yet to be fully elucidated, factors at play include both hereditary risk and environmental influences [40]. The possible link between autoimmunity and the onset of epilepsy has attracted significant interest, and one population-level epidemiological study reported the frequent cooccurrence of epilepsy and certain autoimmune diseases [10]. Although epilepsy has a prevalence of 0.4% in a typical population, its prevalence among individuals with autoimmune conditions is as high as 17.5% [41]. Hence, a significant number of epilepsy cases are autoimmune-related, as emphasised by observational studies that suggested that seizures may be controlled via immunotherapy [42]. The role of specific autoimmune mechanisms and their related pathogenic autoantibodies in seizures has therefore been subjected to increased scrutiny [11, 43]. The possibility of autoimmune-related epilepsy must be carefully considered, particularly in drug-resistant and difficult-to-treat cases [44]. This is any form of epilepsy involving clinical symptoms suggestive of pathogenic involvement of the immune system in the onset of seizures or in the development of neuronal injury after the seizure [45]. This condition characteristically affects otherwise-healthy children and is typified by the sudden onset of one or more of the following: cognitive deterioration, encephalopathy, focal seizures, or other focal neurological defects [46–48]. Additional research is needed to provide an enhanced understanding of the pathogenic mechanisms involved, to identify the ideal immunotherapy and to estimate the prognostic impact of treatment.

The involvement of the gut microbiota in the pathogenicity of autoimmune conditions has been clearly demonstrated [49]. The gut microbiota can be both beneficial and harmful to the host [50]. They are essential for digestive processes and for maintaining homeostasis but are also involved in the development of autoimmune diseases through their function in controlling both the anti- and proinflammatory immune responses [51, 52]. Thus, the immune system and the gut microbiota are intimately linked and simultaneously affect each other [53]. Moreover, modulation of the immune system by the microbiota involves not only the intestinal environment but also the nervous system [54]. Hence, recent studies have examined the involvement of the peripheral and CNS-resident immune pathways in microbiota-gut-brain communication in healthy individuals and in those with neurological disorders [9, 54, 55].

Based on the crosstalk between autoimmunity, the onset of epilepsy, and microbiota, it is feasible that the constitution of the gut microbiota population could influence both the individual's susceptibility to epilepsy and the subsequent progression of the disease [15, 56]. Experimental research has indicated a significant link between the onset of epilepsy and elevated levels of proinflammatory cytokines such as IL-6 and IL-1*β* [57, 58], and it is well proven that cytokines are a key to driving and regulating human Th17 responses [59]. Hence, the onset of autoimmune-linked epilepsy could be triggered by commensal microbiota via spontaneous secretion of proinflammatory cytokines leading to an increase in Th17 cells [60].

#### 2.2.2. Recent Studies of Epilepsy Involving the Gut Microbiota

Although investigations into the link between epilepsy and the intestinal microbiota remain in their infancy [61], Table 1 presents a number of studies with published results.

For instance, the 16s ribosomal DNA obtained from faecal samples was subjected to high-throughput sequencing to examine the microbiome compositions of 49 drug-sensitive epilepsy patients, 42 drug-resistant epilepsy patients, and a control group of 65 healthy individuals [62]. The gut microbial population of the drug-resistant individuals was found to be considerably altered relative to that of the control group, with anomalously raised levels of uncommon flora [62]. Moreover, elevated levels of *Lactobacillus* and *Bifidobacteria* were noted in individuals with fewer seizures (a maximum of four seizures in 1 year). Meanwhile, the drug-sensitive patients displayed gut microbiome populations comparable to those of the control group. These results indicate the possible involvement of dysbiosis in the development of drug-resistant epilepsy; hence, a novel approach to the treatment of drug-resistant epilepsy might involve restoration of a healthy gut microbial population [62].

Another recent study examined whether rats can be made susceptible to epilepsy by induced dysbiosis resulting from chronic restraint stress [63]. Progression of the illness was accelerated, and the duration of the resulting seizures increased, not only in chronically stressed individuals but also in so-called sham-stressed rats (previously healthy rats given a transplanted microbiome population from stressed individuals) [63]. In addition, the proepileptic impacts of restraint stress were reversed when the chronically stressed individuals were given a transplanted microbiome from the sham-stressed rats [63]. These observations provide direct evidence for a link between alterations in the gut microbiome (specifically caused by chronic stress) and an enhanced predisposition to epilepsy [63].

Although the clinical application of microbiota in the treatment of brain diseases has not been widely investigated, one strategy with positive potential is restoration of the gut microbiota via faecal microbiota transplantation (FMT) [67]. This approach was recently investigated in a case study involving the treatment of long-term (17-year) epilepsy in a patient with Crohn's disease (CD) [64]. During the 20-month follow-up period, the FMT treatment was effective in both alleviating intestinal symptoms and preventing the recurrence of seizures after the withdrawal of antiepileptic drugs [68]. These observations emphasise the contribution of the microbiota-gut-brain axis and support the novel concept of remodeling the gut microbiota in the treatment for epilepsy.

In another contemporary study, the effects of antibiotics upon the frequency of seizures were investigated in six interesting drug-resistant epilepsy cases [65]. Short-term (2-week) cessation of seizures was observed after the antibiotic treatments, again demonstrating the probable role of the gut microbiota in the development and symptoms of epilepsy and supporting the idea that drug-resistant cases could be treated by adjusting the gut microbiota to disrupt the unfavourable microbiota [65]. It implies that such an intervention could directly affect the frequency of seizures by influencing the gut-brain interactions.

A group of 45 drug-resistant epileptic patients were given a 4-month course of probiotics in a prospective study in which the levels of interleukin 6, *γ*-aminobutyric acid, and CD-14 were evaluated along with quality of life (QOLIE-10) and the number of seizures before and after the treatment [66]. Intention-to-treat analysis demonstrated the 50% reduction in seizures required by clinical trials in 28.9% of patients, along with a notable improvement in quality of life [66]. These results demonstrate the potential use of probiotics as a safe supplementary treatment to enhance control of seizures, and hence the quality of life, in patients with drug-resistant epilepsy.

## 3. Links between the Ketogenic Diet, Microbiota, and Epilepsy

### 3.1. Dietary Adjustment of Gut Microbiota

Numerous studies have shown that the composition of the diet is a key factor in determining the composition of the gut microbiome in various conditions of health and at various stages of life [69]. Indeed, changes in diet seem to have the greatest influence upon the gut microbiota, being responsible for much of the general variation in the gut microbiota structure as well as modifying disease susceptibility by either triggering or circumventing dysbiosis [70–72]. The adjustment of gut microbiota via dietary composition primarily involves altering the proportion of dietary fibre, also referred to as microbiota-accessible carbohydrates (MACs), along with dietary protein and fat [73]. The gut microbiota is dependent upon dietary fibre for energy and sustenance. Moreover, the composition of the diet, especially fibre, seems to be a key factor in gut bacterial function, ecology, and diversity [74]. In addition, dietary protein provides the primary source of nitrogen required for the growth of colonic microbes and is critical for carbohydrate assimilation and generation of valuable molecules [75], such as short-chain fatty acids (SCFAs) [76]. However, diets that are high in protein have also been found to raise the levels of harmful metabolites in faeces and are linked to conditions such as cancer and inflammatory bowel disease [77]. Finally, the fat content provides the diet with a high caloric value; 40% to 55% of calories in the Western diet are provided by lipids. It has been shown that the diurnal structural and functional characteristics of the gut microbiota are influenced by a high-fat diet [78]. In addition, it was recently shown that alteration of the gut microbiota by *ω*-3 fatty acids can reduce chronic inflammation and prevent weight gain [79].

### 3.2. Recent Advances in the Treatment of Epilepsy by Ketogenic Diet

The ketogenic diet (KD) restricts the caloric intake to 10% to 25% and consists of more than 90% fat, almost no sugar, and just sufficient amounts of vitamins, minerals, and proteins [80]. Despite the known harmful effects of excess dietary fat, the KD has been used as a therapeutic diet since the early 20^th^ century, and its application in the treatment of epilepsy was inspired by age-old observations regarding the positive effects of fasting [20]. The therapeutic use of the KD has regained popularity in recent years to become the focus of considerable scientific investigation [81]. To increase adherence to the diet, the modern KD has been adapted according to scientifically established variations that make it more appetising and less restrictive (e.g., the modified Atkins diet and the low glycaemic index diet) [82]. The antiepileptic effects of these KD-type diets are presently used in the treatment of drug-resistant adult and child patients [6, 83–87]. In addition to their benefit for certain forms of epilepsy, the KD appears to have some positive effects on other neurological conditions, such as migraine, glaucoma, multiple sclerosis, Parkinson's disease, and Alzheimer's disease [81, 88–90].

Although the positive effects of the KD in reducing epileptic seizures are well proven, the precise mechanism by which this occurs has yet to be fully elucidated. Nevertheless, the recent literature indicates some advances in this topic [87, 91, 92], including the implication of the gut microbiota alongside changes in the functioning of the mitochondria, alteration of neurotransmitter release and neuron function by ketone bodies, and antiepileptic effects of glucose stabilisation and/or fatty acids [91, 93]. Nevertheless, more research is needed to increase our understanding of these potential mechanisms [94].

### 3.3. Involvement of Gut Microbiota in the Treatment of Epilepsy by KD

**Figure 1:**
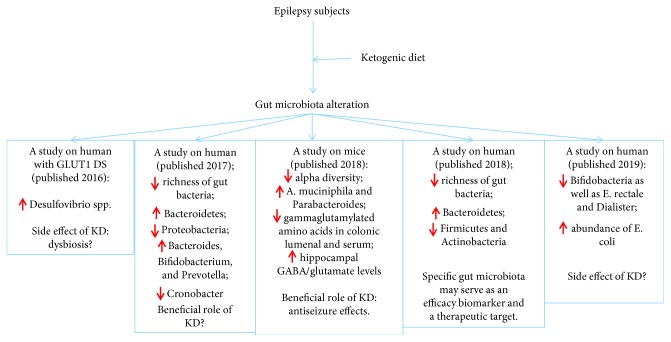
Possible role of gut microbiota in recent studies on the effects of the ketogenic diet on epilepsy patients. GLUT1 DS: Glucose Transporter 1 Deficiency Syndrome; KD: ketogenic diet.

Few studies have dealt with the involvement of the gut microbiota in the treatment of epilepsy with the KD [93, 95]. Examination of the composition and characteristics of the gut microbiota during KD treatment of epilepsy has indicated the potential mechanism presented in Figure 1.

Remarkable findings relating to the microbiota- and ketogenic diet-dependent protection from seizures were reported by Olson *et al.* after a study in which two mouse models of refractory epilepsy were used to demonstrate mediation of the protective effect by the gut microbiota [95]. The susceptibility to and incidence of seizures were found to increase in individuals on the KD diet when given a high-dose antibiotic treatment that led to depletion of the microbiota. However, the negative effect of the antibiotic treatment was then reversed as the gut was recolonised with bacteria. The diversity of gut microbiota was decreased, whilst the relative abundance of *Parabacteroides* and *Akkermansia muciniphila* increased, during the KD diet; hence, these specific changes may play a part in the protection from seizure activity observed. Furthermore, these changes were linked to increased bulk and glutamate levels of gamma-aminobutyric acid (GABA) in the hippocampus.

Following on from this, Hampton suggested that the antiseizure effects of the KD could be attributed to gut microbes. This study was the first to demonstrate the direct involvement of the microbiota in providing the antiseizure effects of the KD in mice [96]. This study also revealed the potential cellular and molecular mechanisms by which interactions between specific bacteria modulate the peripheral metabolites that influence the levels of hippocampal neurotransmitters [96]. Further research is warranted to establish whether the KD leads to comparable effects upon brain metabolites and amino acids in humans and to answer other intriguing questions, such as whether the role of the hippocampus in childhood epilepsy is comparable in mice and in humans [21]. The study's findings could also be relevant to other conditions that respond to the KD, such as Alzheimer's disease, cancer, autism spectrum disorder, and metabolic syndrome [93, 97, 98]. Meanwhile, a study of the effects of the KD upon children with drug-resistant epilepsy indicated a decrease in the diversity of gut microbiota after 1 week, with a decrease in the levels of the phylum Proteobacteria and an increase in the levels of the phylum Bacteroidetes [61]. At the genus level, a decrease in Cronobacter was noted alongside increases in *Prevotella*, *Bifidobacterium*, and *Bacteroides*. The microbiota in epileptic infants was found to differ from that of healthy controls and was also shown to change significantly, with increases in beneficial bacteria and decreases in pathogenic bacteria, in response to the KD [61]. Hence, the study suggested that the KD could quickly modify the gut microbiota and reduce the frequency of seizures in infants with drug-resistant epilepsy.

In a separate investigation, the faecal microbiota profiles of children with resistant epilepsy revealed decreased diversity, with decreased levels of Firmicutes and increased levels of Bacteroidetes after 6 months of KD treatment [99]. The subjects of this study showed various seizure-reduction responses, and those who failed to respond had enhanced levels of *Alistipes*, *Clostridiales*, *Lachnospiraceae*, *Ruminococcaceae*, and *Rikenellaceae* relative to those who responded to treatment [99]. This suggests that the KD may have varied efficacy with respect to altering the composition of the gut microbiota and that specific microbiota may provide both possible therapeutic targets and biomarkers for the efficacy of the treatment in individuals with resistant epilepsy [99].

The above-mentioned study was critiqued by E. Spinelli and R. Blackford, who raised the following questions:

- How does the gut bacteria influence the onset of epilepsy
- Is it possible to use the composition of the gut microbiota as a marker to monitor the effectiveness of treatment, as has been done in mouse models
- Can the bacterial composition be modified as a therapeutic approach

These commenters also point to the need for a large-scale multicentre investigation to build upon the above observations and to better demonstrate whether a possible microbe-based treatment is a rational choice in paediatric refractory epilepsy [100].

In another recent study, six individuals with GLUT1 deficiency syndrome were asked to collect faecal samples before and after 3 months of KD treatment to compare the microbiota compositions [101]. *Bacteroidetes*, *Bifidobacterium spp.*, *Clostridium cluster* XIV, *Clostridium perfringens*, *Desulfovibrio spp.*, *Enterobacteriaceae*, *Faecalibacterium prausnitzii*, *Firmicutes*, and *Lactobacillus spp.* were quantified by reverse-transcription polymerase chain reaction (RT-PCR). The faecal microbial profiles demonstrated statistically significant enhancement in the levels of *Desulfovibrio spp.*, a group of bacteria believed to contribute to aggravated inflammation of the gut mucosa resulting from ingestion of animal fats [101].

In view of the above research, a future prospective investigation on alterations in the gut microbiota of epileptic children subjected to KD treatment is warranted. In addition, it may be logical to propose an empirical test of the potential for prebiotics or probiotics to reestablish the optimum ecological balance of the intestinal microbiota in individuals whose faecal samples indicate dysbiosis [102].

A study in 12 children with drug-resistant epilepsy was recently published online [103]. Faecal samples were collected before and after 3 months of KD treatment, whilst the parents functioned as a diet control group. Alterations in both the taxonomic and functional profiles were detected via shotgun metagenomic DNA sequencing. The treatment resulted in notable reductions in the relative abundances of *Bifidobacteria*, *Dialister*, and *Eubacterium rectale* along with an increase in the relative abundance of *Escherichia coli* [103]. Alterations in 29 SEED subsystems were indicated by functional analysis, including the decline of seven carbohydrate metabolism pathways. The analysis demonstrated that the *Bifidobacteria* and *E. coli* contributed significantly to the functional changes [103]. The study expressed misgivings surrounding the effects of the KD upon the gut microbiota and the patients' general health because the relative abundance of beneficial fibre-consuming bacteria decreased in response to the KD treatment. Consequently, additional research is needed to establish whether these specific alterations are essential for the therapeutic impact of KD treatment.

The KD incorporates a range of mechanisms that lead to decreased neuronal excitability, including alteration of the gut microbiota [104]. Further classification of the specific mechanisms could contribute to the replacement of the strict KD treatment by dietary supplements, such as probiotics and/or prebiotics. Moreover, recognition of the disease-altering characteristics of KD therapy could provide hope for a long-term therapeutic effect that could continue even after the diet has ended.

## 4. Outlook

Recent studies have demonstrated a close link between epilepsy and the gut microbiome. Moreover, the mechanism behind the antiseizure effects of the KD in epileptic patients may be contributed by the gut microbiota. There is therefore much potential in optimising the KD to promote specific microbes as a neuroprotective treatment for drug-resistant epilepsy. Nevertheless, numerous questions must still be fully addressed, including the specific mechanism by which the gut microbiota influences the onset of epilepsy and the possible clinical applications of findings relating to the function of gut microbiota in the KD treatment of epilepsy. Moreover, these questions can only be answered with the establishment of large multicentre research efforts. The KD has potential benefits not only in epilepsy but also in numerous other disorders linked to changes in GABA, including Alzheimer's disease, anxiety, autism, Parkinson's disease, and schizophrenia. Hence, a broad spectrum of grave health issues could be positively affected by the development of a neuroprotective therapy involving microbes.

## Figures and Tables

**Table 1:** Main findings of studies on gut microbiota and epilepsy.

Subjects	Age	Population	Methodology	Findings	Year	Authors
Human	5-50 years old	Drug-resistant epilepsy (*n* = 42), drug-sensitive epilepsy (*n* = 49), and healthy control (*n* = 65).	16s rRNA-based metagenomics	An abnormally increased abundance of rare flora. Bifidobacteria↑ and Lactobacillus↑ in patients with fewer seizures (no more than 4 seizures per year).	2018	Peng *et al.* [62]
Sprague-Dawley rats	45 days old	Chronic-stressed rats and sham-stressedrats.	Faecal microbiota transplantation (FMT) to recipients, in which commensal microbiota had been depleted by antibiotics	Perturbations in the gut microbiome, particularly those associated with chronic stress, in those with increased susceptibility to epilepsy.	2018	Medel-Matus *et al.* [63]
Human	22 years old	A girl with Crohn's disease (CD) and a 17-year history of epilepsy.	Faecal microbiota transplantation (FMT)	FMT achieved remission of intestinal and neurological symptoms in a girl with CD and a 17-year history of epilepsy. The finding inspires a novel treatment for epilepsy through remodeling of the gut microbiome.	2017	He *et al*. [64]
Human	10-16 years old	Six patients with drug-resistant epilepsy.	Antibiotic treatments	Patients attained temporary seizure freedom during antibiotic treatment.	2018	Braakman and van Ingen [65]
Human	Mean age 44 years old	45 patients with drug-resistant epilepsy.	Probiotic treatments	28.9% of all patients displayed a greater than 50% reduction in the number of seizures. A significant improvement was observed in patients' quality of life. Probiotics may be an option for supplementary therapy.	2018	Gomez-Eguilaz *et al*. [66]
